# Effects of ruminal administration of soy sauce oil on functional fatty acids in the rumen, blood and milk of dairy cows

**DOI:** 10.5713/ajas.19.0913

**Published:** 2020-02-25

**Authors:** Daiji Konno, Masanobu Takahashi, Ikuo Osaka, Takenori Orihashi, Kiyotaka Sakai, Kenji Sera, Yoshiaki Obara, Yasuo Kobayashi

**Affiliations:** 1 Graduate School of Agriculture, Hokkaido University, Sapporo, Hokkaido 060-8589, Japan; 2 Dairy Research Center, Hokkaido Research Organization, Nakashibetsu, Hokkaido 086-1135, Japan; 3 Mito Research Center, Meiji Feed CO., LTD., Ibaraki, Ibaraki, 311-3123, Japan

**Keywords:** Dairy Cow, Fatty Acid, Milk, Rumenl Soy Sauce Oil

## Abstract

**Objective:**

Soy sauce oil, a byproduct of whole soybean processing by the soy sauce industry, was evaluated as a source of linoleic acid for dairy cows for the purpose of manipulating the composition of milk.

**Methods:**

Eight dairy Holstein cows fitted with rumen cannulas were used for ruminal administration of soy sauce oil for a 28-day period using a 4×4 Latin square study design with 4 doses (0, 200, 400, and 600 g soy sauce oil/d).

**Results:**

Although dry matter intake and milk yield were not affected by soy sauce oil administration, ruminal concentrations of total volatile fatty acids and acetate were decreased, specifically at 600 g/d administration. While milk fat percentage was decreased with administration of soy sauce oil, proportions of linoleic, vaccenic and conjugated linoleic acids in the rumen, blood and milk were increased with increasing soy sauce oil dose.

**Conclusion:**

These results suggest that soy sauce oil feeding could be useful for improving milk functionality without adverse effects on animal production performance when fed at less than 400 g/d.

## INTRODUCTION

Milk production has been reconsidered in terms of its function; in particular the biological importance of the milk fatty acid *cis-9 trans-11* conjugated linoleic acid (CLA) has received increased scrutiny since the early 2000s [1]. Since CLA is thought to have potential anti-atherogenic, anti-obesity, and anti-carcinogenic roles [2,3], it is expected to impart added value to milk. As CLA is mainly synthesized in the rumen through the bio-hydrogenation process of linoleic acid, attempts to increase CLA production have been made by feeding oils rich in linoleic acid, such as soybean oil [4] and extruded soybean [5], to dairy cows.

On the other hand, feeding oil could negatively impact dry matter intake (DMI) due to decreased gut hormone secretion, oxidation of liver fat [6], depression of rumen fermentation [7,8], and decreased fiber digestibility [9]. Furthermore, it is known that increasing the amount of free unsaturated fatty acid or esterified unsaturated fatty acid intake is likely to decrease milk fat [10]. Therefore, dosing is a crucial consideration for the proper application of feeding oil to dairy cows.

Soy sauce oil, a by-product of whole soybean processing by the soy sauce industry, is the oil fraction separated after compressing whole soy sauce mash. The fatty acid composition of soy sauce oil is similar to that of soybean oil and mainly consists of linoleic acid [11]. Therefore, this oil byproduct represents a promising functional feed resource for farm animals. Prior to the first attempt to explore applications in dairy cattle nutrition by Shibata et al [12], the majority of soy sauce oil had been applied for fuel use as an alternative to heavy oil. These authors indicated that while dry mater intake was not affected, ruminal concentrations of total volatile fatty acids (VFA) and milk fat percentage were decreased, and the proportion of CLA in milk fat was increased following feeding of 400 g/d soy sauce oil. We also attempted a larger amount of soy sauce oil administration into the rumen of dairy cows (1 kg/d) and found 5.9 to 8.8 times increase of CLA in milk [13]. These results suggest the soy sauce oil has the functionality to improve milk quality in terms of CLA richness. In addition, use of such byproducts can contribute to not only improvement of resource recycling, but also higher feed self-sufficiency. However, as the above studies were performed at a single dosing level of soy sauce oil, the optimal level remains to be experimentally defined.

Therefore, to clarify the suitable range of soy sauce oil feeding, we carried out an experiment in which different doses of soy sauce oil were ruminally administered to dairy cows. The application of soy sauce oil to dairy feed is discussed in the context of the effects of soy sauce oil administration on DMI, ruminal fermentation and milk production.

## MATERIALS AND METHODS

### Animals and diets

The research protocols regarding animal care followed the Guidelines for Animal Experimentation of the local independent administrative agency of the Hokkaido Research Organization.

Eight primiparous Holstein cows (574±29 kg body weight with 116±22 days in milk) were used in the present study. Cows were housed in tie stalls with rubber mats bedded with sawdust. The animals had been previously fitted with rumen cannulas (soft plastic cannulas with a 10 cm internal diameter; Bar Diamond Inc., Parma, ID, USA). The experiment was carried out using a 4×4 Latin square design with a 28-day treatment period, in which the first 21 days were for diet adaptation and the last 7 days were for sampling and data collection. Cows were randomly assigned to one of four treatment groups: 0, 200, 400, or 600 g/d soy source oil administration. Soy sauce oil was administered into the rumen via the cannula twice daily (0830 and 1730 h) in two equal doses.

Ingredients and chemical composition of diet used in the present study are shown in Table 1. The animals were given a diet of total mixed ration (TMR) containing 50.0% grass (mainly Timothy) silage, 37.0% corn, 11.0% soybean meal and 2.0% di-calcium phosphate on a dry matter (DM) basis. The DM content of grass silage was determined weekly to adjust the diet formulation on an as-fed basis. Cows were fed TMR once daily (10:00) *ad libitum*, which was equivalent to 110% of the expected intake. The amounts of diet offered and refused were recorded daily. Fresh water was available at all times. Cows were milked twice daily (0900 and 1900 h) and milk production was recorded at each milking. Total digestible nutrient (TDN) requirement was calculated based on body weight and milk production of each cow [14] and TDN sufficiency level was calculated.

### Sample collection

Cows were weighed at the end of each treatment period. Milk was sampled on 4 consecutive days during the collection period and pooled to obtain representative samples. An aliquot of the milk sample was placed into plastic vials and stored at −30°C for long chain fatty acid analysis.

Rumen and blood samples were obtained on day 28 via the ruminal cannula and jugular vein catheter at −0.5, 0, 0.5, 1, 2, 3, 4, 5, 6, 7, 8, and 9 h after the morning administration of soy sauce oil (to more dynamically know metabolic responses to oil administration, these frequent samplings were conducted). The blood catheter was inserted 12 h before the first sampling and removed just after the last sampling of each sampling day. Rumen samples were taken from the dorsal and ventral sac by hand, mixed, and then strained through 4 layers of surgical gauze to obtain representative filtrates in a plastic vial. The filtrates were used for pH measurement using a HM-50G electrode (TOA-DKK, Tokyo, Japan), and then stored at −30°C for further analysis. Blood samples were centrifuged to obtain serum and stored at −30°C until analysis.

Feed samples including grass silage, corn and soybean meal were collected in each period and dried in a 60°C forced air oven for 48 h for DM determination. The dried feed samples were ground and sieved through a 1-mm screen using a cutting mill and used for chemical analysis.

### Chemical and statistical analysis

Feed samples were analyzed for DM, crude protein (CP), ether extract (EE) and neutral detergent fiber using standard procedures [15]. Fat, protein and lactose levels in milk samples were assessed using an infrared analyzer (Milko-Scan FT120, Foss Electric, Hillerod, Denmark).

Frozen rumen, milk and blood serum samples were thawed at 4°C and assessed using the following chemical analyses. Ammonia nitrogen was analyzed using the phenol hypochlorite method [16]. VFA was analyzed using a gas chromatograph (GC-14B; Shimadzu Co., Kyoto, Japan) with crotonic acid as an internal standard [17].

Fatty acid analysis was performed after conversion of fatty acids into fatty acid methyl esters (FAME), with different protocols used for milk and other samples (rumen and blood serum) as follows. Milk fatty acids were analyzed according to the method of Aii et al [18] with minor modifications. Briefly, after milk fat was extracted with hexane, FAME were prepared by the trans-methylation procedure according to the method of Christie [19]. Fatty acids of rumen and serum samples were analyzed according to the modified liquid-liquid extraction method, in which sodium hydroxide methanol and boron tri-fluoride methanol reagents were used for conversion to FAME. Methyl esterified samples were analyzed using the GC-14B gas chromatograph (Shimadzu Co., Japan) fitted with a flame ionization detector, using a silica capillary column (SP-2560, 100 m×0.25 mm (i.d.) with 0.20 μm film thickness; Spelco, Bellefonte, PA, USA).

Data were analyzed using JMP (SAS Institute Inc., Cary, NC, USA) as a replicated 4×4 Latin square design. When the fixed effect was significant, the difference among means was determined with Tukey’s multiple comparison test. Linear and quadratic effects were also evaluated through administration levels. A level of p<0.05 was defined as significant.

## RESULTS

Table 2 shows the changes in nutrient intake following ruminal administration of soy sauce oil. With increased doses of soy sauce oil, total DMI and grass silage intake/total DMI were linearly reduced. While EE intake/total DMI and TDN intake/total DMI were linearly increased, CP intake/total DMI was not changed. TDN requirement, intake and sufficiency level were not different between oil administration levels.

Table 3 presents milk yield and composition in cows ruminally administered soy sauce oil. Milk yield was unaffected by soy sauce oil intake, ranging from 24.6 to 26.0 kg/d. In addition, fat-corrected-milk (FCM) yield was also not affected, although a slightly lower value was observed in cows receiving the highest dose of soy sauce oil (600 g/d). Milk fat percentage decreased linearly with the administration level of soy sauce oil and the lowest value was observed in cows receiving 600 g/d. Protein, lactose and solids-not-fat contents were not affected by ruminal administration of soy sauce oil.

**Figure 1:**
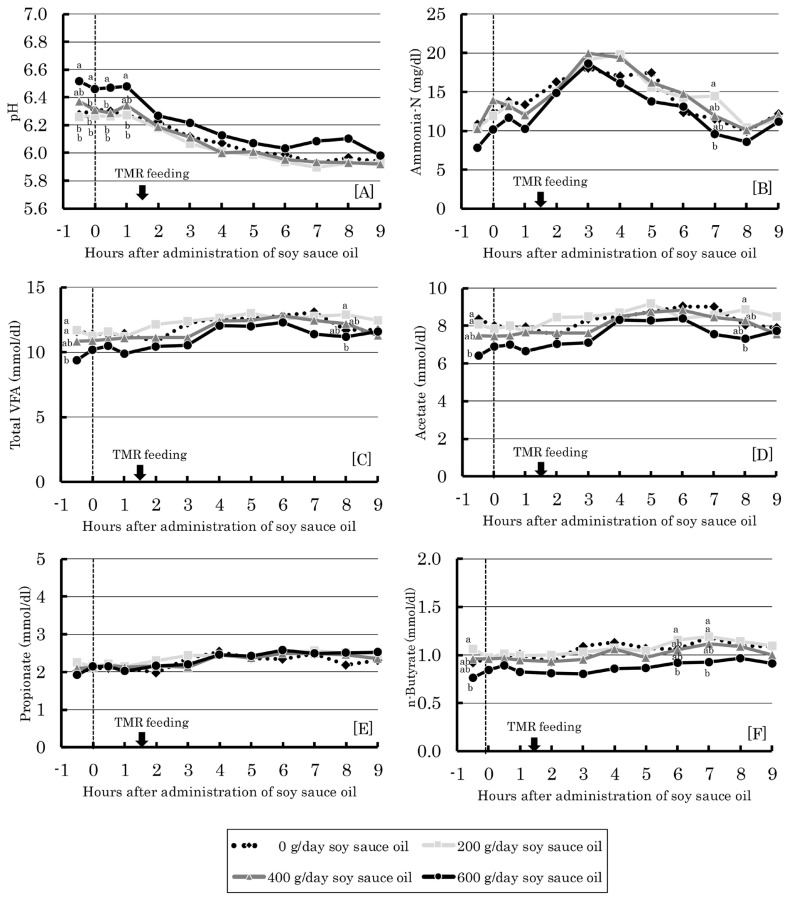
Effect of ruminal administration of soy sauce oil on ruminal pH (A), ruminal concentration of ammonia nitrogen (B), total volatile fatty acid (C), acetate (D), propionate (E) and n-butyrate (F) in dairy cows. ^a,b^ Means with different superscripts differ significantly (p<0.05). Bars represent standard errors.

Ruminal fermentation parameters, including pH, ammonia N, and VFA, as affected by administration of soy sauce oil are shown in Figure 1. Although ruminal pH, ammonia, concentrations of total VFA, acetate, and butyrate showed almost similar changes following ruminal administration of soy sauce oil, the values at the highest administration level (600 g/d) were consistently lower than those observed at other administration levels of soy sauce oil (except for pH, which showed consistently higher values). In particular, the acetic acid concentration with 600 g/d soy sauce oil was always lower throughout the sampling period. The decrease of the pooled value for acetate concentration across sampling points was 10.8%. Propionate concentration did not show any particular change with soy sauce administration. Thus, except for at 600 g/d, soy sauce oil administration did not negatively affect rumen fermentation.

**Figure 2:**
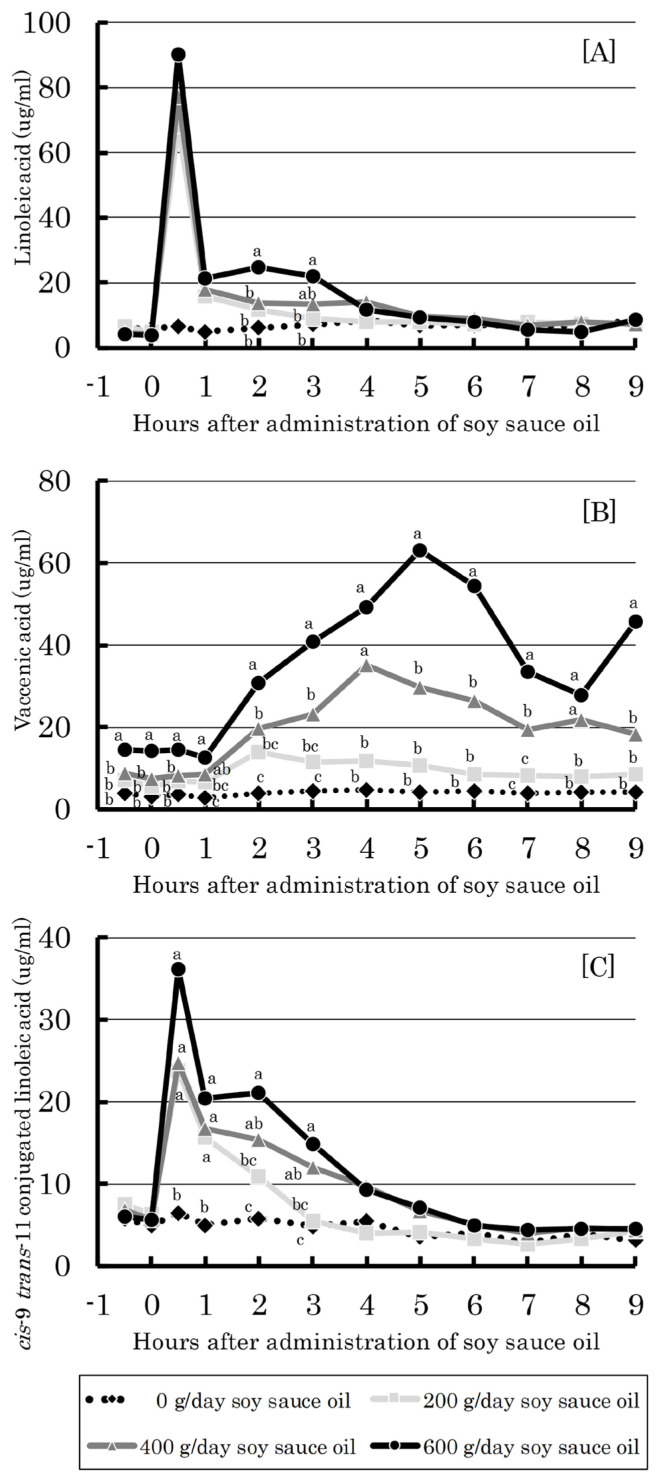
Effect of ruminal administration of soy sauce oil on ruminal concentration of linoleic acid (A), vaccenic acid (B), and cis-9 trans-11 conjugated linoleic acid (C) in dairy cows. ^a–c^ Means with different superscripts differ significantly (p<0.05). Bars represent standard errors.

Ruminal concentrations of linoleic acid, *trans-11* vaccenic acid (vaccenic acid) and CLA in cows administered soy sauce oil at different levels are shown in Figure 2. All cows showed the maximum value of each fatty acid after administration as follows: 0.5 h for linoleic acid, 4 to 5 h for vaccenic acid and 0.5 h for CLA. In addition, the maximum concentration of each fatty acid increased with increasing administration level. Linear effects were observed (p<0.01) for dose responses in all the above fatty acids.

**Figure 3:**
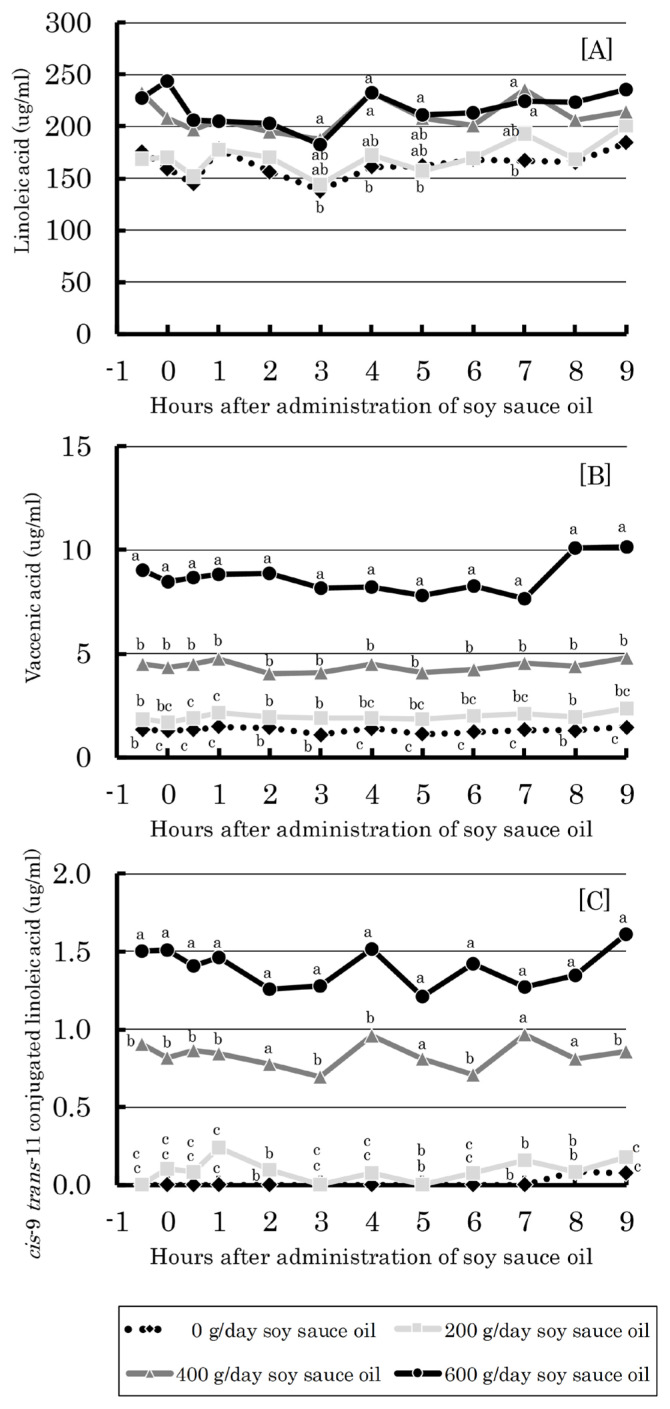
Effect of ruminal administration of soy sauce oil on blood serum concentration of linoleic acid (A), vaccenic acid (B), and cis-9 trans-11 conjugated linoleic acid (C) in dairy cows. ^a–c^ Means with different superscripts differ significantly (p<0.05). Bars represent standard errors.

Concentrations of linoleic acid, vaccenic acid and CLA in blood serum of cows administered soy sauce oil are shown in Figure 3. The concentration of linoleic acid was the highest (ca. 150 to 250 μg/mL), followed by vaccenic acid (ca. 2 to 10 μg/mL) and CLA (ca. 0 to 1.6 μg/mL). All of these acids were influenced by soy sauce oil, and the values increased linearly (p<0.01) with increasing administration level.

**Figure 4:**
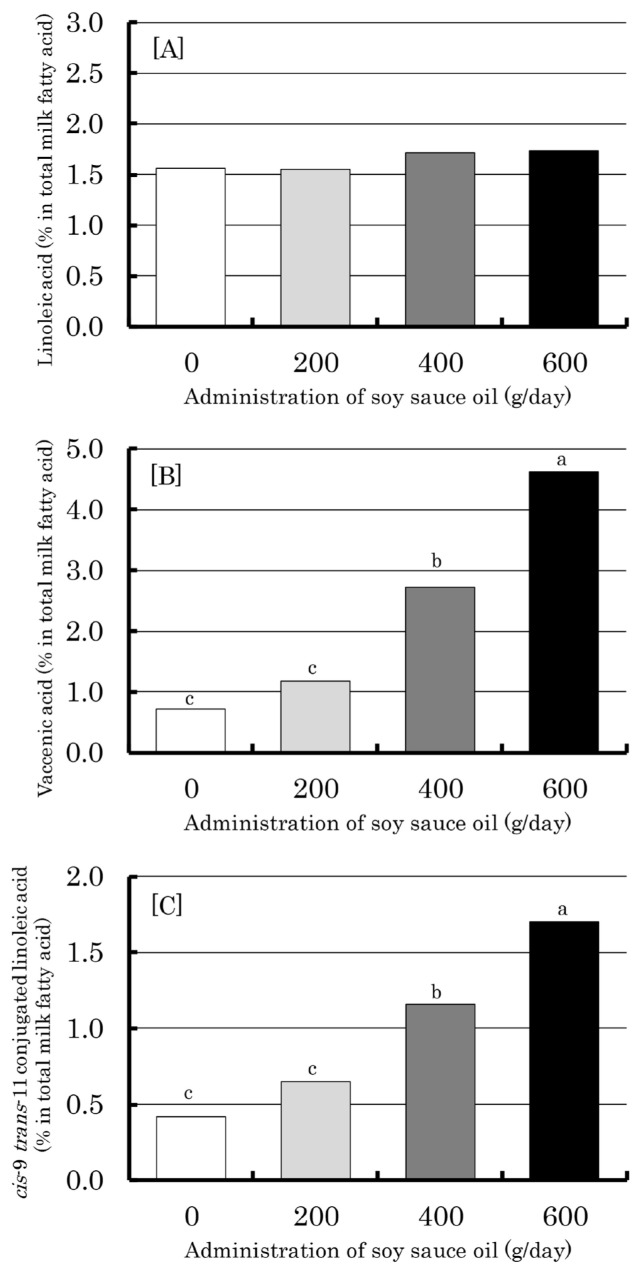
Effect of ruminal administration of soy sauce oil on proportion of linoleic acid (A), vaccenic acid (B), and cis-9 trans-11 conjugated linoleic acid (C) in total milk fatty acid in dairy cows. ^a–c^ Means with different superscripts differ significantly (p<0.05). Bars represent standard errors.

Figure 4 illustrates the proportions of linoleic acid, vaccenic acid and CLA in total fatty acids in milk from cows administered soy sauce oil at different levels. Although the proportion of linoleic acid did not change among the different levels of soy sauce oil administration, the proportions of vaccenic acid and CLA were increased linearly (p<0.01) as the administration level increased, showing higher values at 400 and 600 g/d administration than at 0 and 200 g/d. CLA content in milk (mg/100 g milk) was calculated to be 17.5, 26.0, 45.6, and 57.0 for 0, 200, 400, and 600 g/d administration of soy sauce oil, respectively. Similarly, vaccenic acid contents were 30.4, 47.2, 107.4, and 157.2 (mg/100 g milk), respectively.

## DISCUSSION

New feed materials such as soy sauce oil are expected to have insignificant effects on feed intake, as DMI is an important factor in the production performance of dairy cows. In general, the amount and fatty acid profile of oil, especially the fatty acid composition of digesta reaching the small intestine, are known as determinants of feed intake [20]. In the present study, ruminal administration of soy sauce oil up to 600 g/d did not significantly reduce DMI of dairy cows (Table 2), which is in good agreement with Shibata et al [12], who reported that DMI was not affected by feeding soy sauce oil at 400 g/d. More importantly, milk and FCM yields were unaffected by any level of soy sauce oil administration in the present study (Table 3), although FCM yield was decreased by administration of a larger amount (1 kg/d) of soy sauce oil [13]. These are favorable characteristics for future application of soy sauce oil, even though the milk fat content showed a decreasing tendency with soy sauce oil administration (600 g/d) (Table 3).

Although the decrease of total VFA concentration at 2 h after feeding of soy sauce oil was previously reported Shibata et al [12], no such decrease was observed up to the level of 400 g/d in the present study (Figure 1). The highest administration of soy sauce oil (600 g/d) consistently lowered total VFA, acetate and butyrate concentrations (Figure 1). A large amount of oil (600 g/d) could depress rumen fermentation (as observed in the decrease of total VFA), presumably with depressing microbial activity by oil itself and with inhibiting microbial access to oil-coated feed particles. Rumen microbial community analysis can reveal the detailed mechanism in the utilization of soy sauce oil-supplemented diet. It is generally acknowledged that decreases in acetate and butyrate result in a decrease in milk fat (Table 3) [21]. Thus, feeding soy sauce oil up to 400 g/d is appropriate considering production performance, in relation to feed intake and rumen fermentation, of dairy cows.

The biggest impact of the present study results is the dose-dependent increase in concentration of functional CLA in the rumen, blood and milk with soy sauce oil administration (Figures 2 to 4). This suggests the beneficial influence of soy sauce oil feeding on dairy cows and milk consumers via the health-promoting action of CLA [1]. According to the temporal change of ruminal CLA, the generation of CLA from linoleic acid is rapid (within 1 h after administration) followed by that of vaccenic acid (Figure 2), which occurs through isomerization and bio-hydrogenation by specific rumen bacteria [22]. Meanwhile, the concentrations of CLA, vaccenic acid and linoleic acid in blood serum were fairly stable irrespective of sampling time over the 9.5 h (Figure 3), indicating that these fatty acids can be distributed in whole animal tissues including mammary glands. This could cause elevations of CLA and vaccenic acid in milk in a dose-dependent manner (Figure 4). As vaccenic acid can be converted to CLA by Δ-9 desaturase in mammary glands and other body tissues [23], CLA in milk could be, in part, derived from vaccenic acid, as pointed out by Toral et al [24].

When calculated from the CLA proportion of total fatty acids of milk (Figure 4) and milk fat content (Table 3), the CLA content of milk at the preferred administration level of soy sauce oil (400 g/d) was 45.6 mg/100 g milk. Even though the milk fat yield decreased with the administration of soy sauce oil (Table 3), an increase in CLA in milk was indeed observed in the present study. This confirms that soy sauce oil feeding is anticipated as a means to produce functional milk. There have been many attempts to use supplementary feeds, represented by plant oils, to enhance CLA concentrations in digesta [25,26] and animal products including milk and meat [4,24]. However, soy sauce oil, a byproduct of the soy sauce industry, as evaluated in the present study, could be one of the candidates for a cost-effective approach to increase CLA in milk. Obviously, soy sauce oil should be properly formulated into the diet of cows and evaluated in a feeding study (as opposed to forced administration employed in the present study), in which the feeding level of soy sauce oil is set at a maximum of 400 g/d. Practically, soy sauce oil could be formulated by proper ways easy for handing, e.g. in mash or meal form to which oil is absorbed.

Ruminal supplementation of soy sauce oil can shift fatty acid profiles of rumen content, blood serum and milk toward higher proportions of linoleic acid, vaccenic acid and CLA, depending on the administration level. Daily administration of up to 400 g/cow is proposed to be suitable for this purpose without detrimental effects on VFA and milk fat production.

## Figures and Tables

**Table 1:** Ingredients and chemical composition of diet fed to dairy cows

Items	Total mixed ration	Soy sauce oil
Ingredients (% of dry matter)		
Grass silage	50.0	
Corn	370.	
Soybean meal	11.1	
Di-calcium phosphate	2.0	
Chemical composition (% dry matter)		
Crude protein	15.0	-
Ether extract	4.2	99.7
Neutral detergent fiber	41.3	-
Non fiber carbohydrate1)	33.5	-
Total digestible nutrients	69.9	218.32)

1) Organic matter – (crude protein + ether extract + neutral detergent fiber)

2) Value of vegetable cooking oil from standard tables of feed composition in Japan (National Agriculture and Food Research Organization, 2009).

**Table 2:** Effect of ruminal administration of soy sauce oil on feed intake of dairy cows

Items	Soy sauce oil (g/d)				SEM	Contrast (p-value)1)	
	
	0	200	400	600		Linear	Quadratic
Dry matter intake (kg)	18.1	18.4	17.6	17.0	0.53	0.05	0.09
Grass silage/dry matter intake (%)	50.0	49.5	48.9	48.2	0.04	<0.01	<0.01
Ether extract/dry matter intake (%)	3.9^d^	5.0^c^	6.1^b^	7.3^a^	0.07	<0.01	<0.01
Crude protein/dry matter intake (%)	15.6^a^	15.4^b^	15.3^c^	15.1^d^	0.01	0.09	0.24
Total digestible nutrient/dry matter intake (%)	71.3^d^	73.3^c^	75.0^b^	76.9^a^	0.10	<0.01	<0.01
Requirement of total digestible nutrients (kg)	14.0	14.1	14.2	13.3	0.44	0.28	0.26
Intake of total digestible nutrients (kg)	13.0	13.5	13.2	13.1	0.38	0.99	0.54
Intake/requirement (%)	92.9	95.7	93.0	98.5	2.89	0.19	0.26

SEM, standard error of the mean.

1) Linear and quadratic effect for incremental amount of soy sauce oil administration.

a–d Means with different superscripts differ significantly (p<0.05).

**Table 3:** Effect of ruminal administration of soy sauce oil on milk yield and composition in dairy cows

Items	Soy sauce oil (g/d)				SEM	Contrast (p-value)1)	
	
	0	200	400	600		Linear	Quadratic
Milk yield (kg/d)	24.6	25.4	26.0	24.8	1.25	0.68	0.73
4% fat corrected milk (kg/d)	24.8	25.5	25.3	22.1	1.52	0.15	0.13
Fat (%)	4.20	4.06	3.94	3.36	0.312	0.01	0.02
Protein (%)	3.60	3.52	3.39	3.38	0.114	0.05	0.15
Lactose (%)	4.51	4.53	4.55	4.57	0.070	0.44	0.74
Solid not fat (%)	9.13	9.08	8.98	8.98	0.162	0.30	0.58

SEM, standard error of the mean.

1) Linear and quadratic effect for incremental amount of soy sauce oil administration.
